# A thank you note to our reviewers

**DOI:** 10.1002/jgf2.590

**Published:** 2022-10-27

We would like to express our deepest gratitude to all the individuals who have provided their valuable time and expertise to support Journal of General and Family Medicine. The Editorial Board wishes to acknowledge with gratitude the following Reviewers for reviewing manuscripts during the past year.

Abe, Kazuhiro

Abe, Toshikazu

Akashi, Yusaku

Akiyama, Yutaro

Aoki, Takuya

Asakura, Kentaro

Ayabe, Makoto

Azuma, Teruhisa

Berger, Fabian K

Chen, Yen‐Yuan

Chiba, Toshimi

Fujita, Junko

Fujita, Koji

Fujiwara, Dai

Fukui, Sho

Funakoshi, Hiraku

Hagiya, Hideharu

Halcomb, Elizabeth

Hamada, Shuhei

Hamai, Ayano

Harada, Nahoko

Harada, Taku

Harada, Yukinori

Hase, Ryota

Hasunuma, Naoko

Hayashi, Tetsuro

Hayashi, Tsuneari

Hinata, Yuki

Hirai, Takuya

Hirakawa, Yoshihisa

Hirayama, Yoko

Hirooka, Nobutaka

Hosoda, Tomohiro

Ikemoto, Tatsunori

Imagawa, Atsushi

Imamura, Takeaki

Inata, Yu

Inoue, Kenji

Inoue, Machiko

Isenberg‐Grzeda, Elie

Ishiguro, Kazuya

Ishii, Tadashi

Ishikane, Masahiro

Ishikawa, Yukiko

Ishimaru, Hiroyasu

Isono, Hiroki

Ito, Makoto

Itoh, Naoya

Iwamuro, Masaya

Iwata, Hiroyoshi

Jindai, Kazuaki

Kadono, Mika

Kakeya, Hiroshi

Kameda, Toru

Kameoka, Junichi

Kamimura, Kenya

Kanakubo, Yusuke

Kaneko, Makoto

Kanke, Satoshi

Kanzawa, Yohei

Kataoka, Yuki

Katayama, Kohta

Katayama, Yusuke

Kato, Koki

Kawahara, Hiroaki

Kawashima, Atsushi

Kenzaka, Tsuneaki

Kido, Hidenori

Kijima, Tsunetaka

Kimura, Takuma

Kise, Morito

Kishida, Naoki

Ko, Yura

Kobayashi, Tadashi

Kohashi, Kosuke

Kojima, Taro

Kondo, Takeshi

Kosaka, Shintaro

Kuniya, Toshikazu

Kurano, Makoto

Kurata, Takako

Kutsuna, Satoshi

Little, Sahoko

Maita, Hiroki

Maki, Nobuyuki

Matono, Takashi

Matsumura, Masami

Matsumura, Shinji

Matsuno, Kei

Matsuyama, Yasushi

Midlöv, Patrik

Miyagami, Taiju

Miyahara, Reiko

Miyamichi, Ryosuke

Miyatake, Nobuyuki

Miyazaki, Kei

Mizumoto, Junki

Momosaki, Ryo

Morales, Mignodel M

Morikawa, Toru

Morimoto, Katsuhiko

Moriya, Akinari

Morotomi, Nobuo

Murayama, Shinichi

Nagai, Yuki

Nagano, Ayano

Nagasaki, Kazuya

Nagasawa, Tasuku

Nakai, Kazuo

Nakajima, Shigemi

Nakayama, Kuniko

Narita, Masashi

Narumoto, Keiichiro

Nguyen, Chuyen

Nishimura, Yoshito

Nishizaki, Yuji

Nomura, Kyoko

Nomura, Toshifumi

Nomura, Yoshikatsu

Nomura, Yu

Obika, Mikako

Ohbu, Sadayoshi

Ohira, Yoshiyuki

Ohta, Ryuichi

Oishi, Ai

Okada, Tadao

Omboni, Stefano

Ono, Yuko

Oshima, Kumi

Ozone, Sachiko

Sada, Ryuichi

Sadohara, Michito

Saiki, Takuya

Sakai, Masahiro

Sakamoto, So

Sako, Akahito

Sasaki, Yosuke

Satoi, Yoshinao

Sawaya, Yohei

Sechi, Gian Pietro

Sekimoto, Yasuhito

Sharma, Neeru

Shikino, Kiyoshi

Shimasaki, Teppei

Shimizu, Keiki

Shimizu, Taro

Son, Daisuke

Stepnowsky, Carl J

Sugiyama, Mizuki

Sugiyama, Yoshifumi

Suyama, Yasuhiro

Suzuki, Ryoji

Suzuki, Tomoharu

Takahashi, Fumihiko

Takamura, Akiteru

Takeshima, Taro

Tatsumoto, Muneto

Tetsuka, Syuichi

Tomita, Shiori

Tracy, Marguerite

Tsuchida, Tomoya

Tsuchiya, Norihiko

Tsukuda, Junpei

Ukawa, Shigekazu

Uranaka, Keiichi

Urushibara‐Miyachi, Yuka

Wada, Mikio

Wakabayashi, Hideki

Watanabe, Takamasa

Watanabe, Yukiko

Watari, Takashi

Woodson, B Tucker

Yabuki, Taku

Yahata, Shinsuke

Yamamoto, Shunji

Yamamoto, Yu

Yodoshi, Toshifumi

Yokokawa, Hirohide

Yokoya, Shoji

Yonemoto, Naohiro

Yoshida, Katsunori

Yoshida, Shuhei

Yoshimura, Yoshihiro

Zamani, Nasim

